# Which biosecurity measures are specific to free-range poultry? Insights from a scoping review

**DOI:** 10.1186/s13567-026-01742-w

**Published:** 2026-04-02

**Authors:** Mattias Delpont, Qamer Mahmood, Alberto Sánchez-Cano, Úrsula Höfle, Gerard Eduard Martín-Valls, Alessandra Piccirillo

**Affiliations:** 1 Univ Toulouse, ENVT, INRAE, IHAP, Toulouse, France; 2 Veterinary Epidemiology Unit, Department of Internal Medicine, Reproduction and Population Medicine, Faculty of Veterinary Medicine, Ghent University, Salisburylaan 133, 9820 Merelbeke, Belgium; 3 SaBio Research Group, Institute for Game and Wildlife Research IREC (CSIC-UCLMJCCM), Ciudad Real, Spain; 4 Departament de Sanitat i Anatomia Animals, Facultat de Veterinària, Universitat Autònoma de Barcelona, Travessera dels Turons S/N, 08193 Cerdanyola del Vallès, Spain; 5 Department of Comparative Biomedicine and Food Science, University of Padua, Legnaro, Italy

**Keywords:** Outdoor, prevention, biosecurity, assessment, checklist, free-range, pasture

## Abstract

In poultry farms, the assessment of biosecurity is usually based on checklists designed for production systems that do not allow access to a free-range area. However, providing outdoor access may increase the risk of contamination by certain pathogens (e.g., soil borne pathogens or those transmitted by wild birds). Therefore, in free-range systems, biosecurity assessment should specifically cover practices related to this particular environment. In this scoping review, we aimed to (1) identify and categorize specific biosecurity measures (BSMs) that could be assessed in free-range poultry farms in high-income countries based on published protocols, and (2) describe the context in which these studies were carried out. A systematic search was conducted in four databases (PubMed, Scopus, Agricola, and Web of Science) and reports were screened according to PRISMA-ScR guidelines. Studies were first deduplicated in EndNote™ and then screened in Rayyan. After screening 481 records, 15 studies were retained as eligible for the extraction of contextual data and the list of BSMs specific to free-range poultry systems. The studies relied on the assessment of a heterogeneous number of BSMs, and a total of 52 unique BSMs were identified and subsequently classified into 8 categories and 23 subcategories. Most measures were related to the presence of other species on the outdoor range—mainly wild birds (including attraction, interaction and direct observation), followed by effective range use by poultry. However, some aspects of biosecurity were seldom or only superficially addressed, such as access to the range by workers and equipment, the characteristics of the range, or methods for wild birds scaring. The proposed list and classification of BSMs should provide additional insights into how biosecurity assessment could be adapted and applied in free-range poultry production systems.

**Supplementary Information:**

The online version contains supplementary material available at 10.1186/s13567-026-01742-w.

## Introduction

Over the last few decades, in high-income countries, alternative ways of raising poultry have developed for a variety of reasons, including animal welfare, environmental sustainability, and the reduction of antimicrobial use. As a consequence, a growing number of farms now provide access to an outdoor range [1, 2]. However, free-range poultry may be more exposed to pathogens than poultry raised in confinement [3, 4], and consequently may show higher prevalence rates of various infectious diseases [5–7]. These pathogens may be directly or indirectly transmitted by wild birds and, to a lesser extent, by wild mammals, insects or gastropods [8]. Some pathogens may also persist longer in the outdoor range in resistant forms (spores, oocysts) [9], particularly when they are telluric [10] or due to the difficulty of performing effective cleaning and disinfection on organic matter [11]. In relation to the risk of infection from wild birds, any feature of the outdoor range that increases wild bird attractiveness (e.g., water sources, vegetative cover) may increase the risk of transmission. Moreover, certain aspects of outdoor range management may also introduce additional infection routes, for example through farm equipment entering the outdoor run.

The complex set of strategies (infrastructure, decontamination or compartmentalisation measures, controls) applied to control the risk of infection, persistence, and spread on farms is referred to as biosecurity. Biosecurity should be adapted to the farm type (lay-out, species, production type), epidemiological context (infection pressure, target pathogens and their transmission routes) and economic context (estimated disease consequences). In different contexts, farms may have their biosecurity assessed (through regulations or rules, advisory programs, self-evaluation, or scientific studies) using evaluation tools developed for these purposes (mainly checklists) [12]. In poultry production, however, most tools have been designed for *conventional* production systems (i.e., standardized, integrated, intensive, indoor). Therefore, biosecurity assessment tools may not adequately address the unique challenges (and transmission routes) associated with outdoor access [12, 13].

Therefore, the overall aim of this scoping review (ScR) was to describe the approaches used in research protocols to assess biosecurity measures (BSMs) in free-range poultry farms in high-income countries (and, to some extent, in relatively comparable systems). More specifically, the objectives were to:

- Identify and categorize aspects of biosecurity specific to free-range farming addressed in the literature, by analysing the biosecurity assessment methods used (i.e., on-farm observations, questionnaires);
- Describe the context in which these assessment methods were used (objectives of the studies protocols);
- Discuss, for the identified BSMs, how they can affect infectious disease transmission on farms;
- Identify BSMs of interest for free-range poultry systems that were rarely or never investigated, thereby providing insights into how biosecurity assessment could be improved in these systems.

## Materials and methods

This ScR followed the methodological framework described by Levac et al. [14] and is reported according to the Preferred Reporting Items for Systematic Reviews and Meta-Analyses, extension for Scoping Reviews (PRISMA-ScR) [15].

### Protocol and registration

An a priori protocol was developed, stored in the Padua Research Archive institutional repository [16] and published online on the Systematic Reviews for Animals and Food (SYREAF) website [17].

### Eligibility criteria

The selection of articles followed criteria structured according to the PICo framework (Population, Interest, Context). Population was defined as poultry farms, limited to broiler, layer, turkey, and duck flocks, due to their significance in high-income countries. Interest was the assessment of BSMs in free-range poultry farms. The definition of “free-range” may vary across countries or even be absent. We considered here any production where poultry have access to an area outside an enclosed building, not covered by a roof and with direct access to soil and vegetation. Articles addressing only farm biosecurity (or management issues) without describing the assessment methodology/tools were excluded. Context was defined geographically limited to high-income countries or regions, namely Europe, the United States of America, Canada, Australia, New Zealand, Japan, and Russia. These countries and regions were considered comparable in poultry farming practices and climatic conditions [18]. Only publications in English, French, Spanish, or Italian were included, with no restrictions on year of publication, provided that full-text articles were available. Eligible study types were journal articles reporting original research, specifically observational, cross-sectional, longitudinal, case–control, or cohort studies.

### Information sources

To identify relevant documents, four databases with high recall in biomedical research [19] were searched: Agricola (EBSCO interface) and Web of Science (WoS) (Clarivate interface) available via Baylor University (USA), and Scopus (Elsevier interface) and Medline (PubMed interface) available via Ghent University (Belgium). All WoS databases were searched, including ProQuest™ Dissertation & Theses Citation Index, KCI-Korean Journal Database, Medline, Preprint Citation Index, and SciELO Citation Index, with the exception of Arts & Humanities Citation Index (A&HCI), Conference Proceedings Citation Index-Science (CPCI-S), and Conference Proceedings Citation Index-Social Science & Humanities (CPCI-SSH), as their research focus fell outside the scope of this ScR. The search was conducted on 25 October 2024.

### Search strategy

To maximize sensitivity, a multi-strand search strategy was applied [20], combining the following concepts: [Biosecurity] AND [Outdoor farm] AND [Poultry] AND [Assessment] AND [High-Income countries]. Search terms were standardized across databases but adapted to their specific architectures. The detailed search strategy is presented in Additional file 1.

### Data management

All citations retrieved from the databases were imported into EndNote™ (version 21.0.1) [21] to remove duplicates and retracted articles. The two-phase screening process (title and abstract screening followed by full-text screening) was performed in Rayyan (version 1.6.1) [22]. Rayyan’s AI-assisted functions were used solely as decision-support tools during the screening process. In particular, text annotation and keyword highlighting were used to prioritize records for manual review. All inclusion and exclusion decisions were made by human reviewers based on predefined eligibility criteria.

### Selection process

Citations were screened by six independent reviewers working in pairs to reduce the risk of excluding relevant reports. Each pair screened half of the citations, ensuring that each reference was reviewed independently by two reviewers. Disagreements were resolved by discussion or, if consensus was not reached, by a third reviewer. At the beginning of each phase, a calibration exercise involving all reviewers was conducted on at least 10% of the total number of papers to ensure consistency and resolve disagreements in advance [23].

Eligibility during screening was assessed with the following questions: is the publication language English, French, Spanish, or Italian? Is the full text available? Is the publication an original research article? Is the study concerning broilers, layers, turkeys and ducks? Does the study concern commercial free-range poultry farms? Does the study include biosecurity assessment? Is the study performed in at least one high-income country?

During title and abstract screening, possible answers were “No,” “Maybe,” and “Yes.” A citation was excluded if both reviewers in a pair answered “No” to one of the questions; otherwise, it was retained for full-text screening. At the full-text stage, only studies with “Yes” answers to all questions were included. Reasons for excluding were recorded at this step.

### Data collection process

Four independent reviewers carried out data extraction from the included studies using a Microsoft ExcelⓇ 2016 spreadsheet developed by one reviewer and validated by the group. A calibration exercise was performed before data extraction. After calibration, data was extracted by pairs of reviewers; disagreements were resolved by discussion or by a third reviewer. If necessary, corresponding authors were contacted to clarify uncertainties.

### Data items

Data extracted from eligible studies included: general study information, such as study details (i.e., publication year, authors, journal), geographical location (where the study was conducted), study duration and/or time-frame, study design (cross-sectional, longitudinal, etc.); population data (type of poultry, production system, number of farms, and number of animals per farm); and interest data, such as number of BSMs (specific to outdoor range), tools used to assess biosecurity (questionnaire, observations), and whether quantitative assessment was applied (e.g., biosecurity score, weighting of BSMs). Moreover, all biosecurity-related questions or items included in the studies were extracted.

### Data synthesis

The results of the literature search were comprehensively documented, specifying the total number of citations screened, duplicates removed, and full-text articles assessed for eligibility. A flow diagram was prepared to illustrate the screening process and detail the reasons for exclusion at the full-text stage. A descriptive analysis of the included studies was conducted and presented in tables and figures. In addition, measures were reported in their original wording (when available) or rephrased, and then organized into BSMs, categories, and subcategories.

## Results

### Study selection

**Figure 1:**
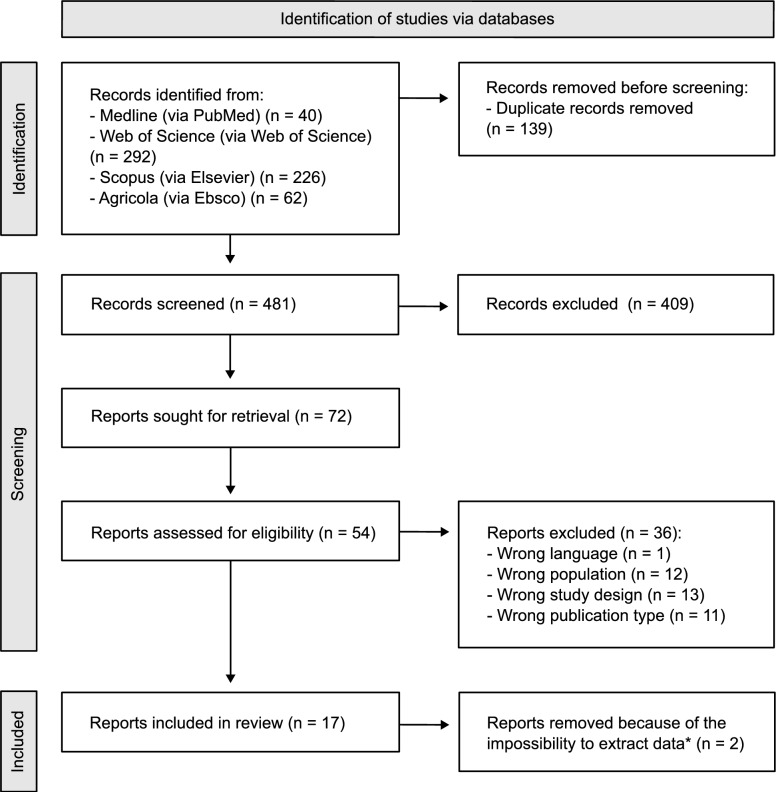
**PRISMA flow diagram showing the study selection process.** The figure presents the selection process for the ScR on biosecurity measures in free-range poultry farms in high-income countries. *: extracted data refers to the name of the biosecurity measures or to the question used to assess them.

The search across the four databases identified 620 articles. After removal of duplicates, 481 unique records remained for the two-phase screening. During title and abstract screening, 72 articles were retained, while 17 met the inclusion criteria at the full-text stage. The main reasons for exclusion were wrong study design (*n* = 13), wrong population (*n* = 12) and wrong publication type (*n* = 11). Out of the 17 articles, BSMs could not be extracted from two, leaving a final set of 15 studies included for data extraction. The workflow of the selection process is presented in Figure 1**.**

### Study characteristics

**Figure 2:**
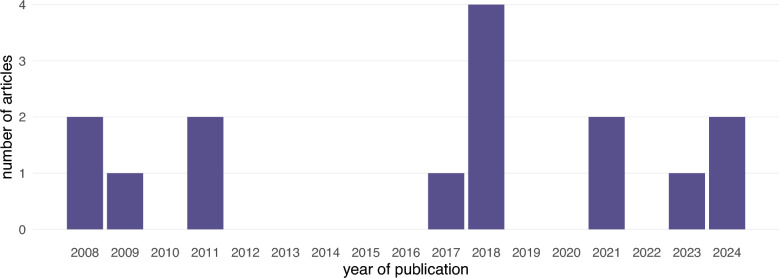
**Year of publication of the 15 articles retained for the analysis of the biosecurity measures specific to free-range poultry farms.**

A total of 15 articles was included for data extraction. All were written in English. Six studies were conducted in Europe, four in Australia, three in North America, and two in the United Kingdom. Two of the European studies covered up to twelve countries. All studies were published between 2008 and 2024 (Figure 2). The main characteristics of the studies are presented in Tables 1 and 2 and the original dataset is provided in Additional file 2.

**Table 1:** **Studies included in the analysis of biosecurity measures specific to free-range poultry farms: location, farm type, and number of farms**

Article reference	Countries*	Poultry category, for free-range	Production type	Number of farms***
[42]	Belgium, France, The Netherlands, Italy, Spain, Poland, Hungary, Finland, Sweden, Serbia, Portugal and Germany	Broilers, layers	Free-range + confined	50
[47]	United Kingdom	Broilers	Free-range + confined	2
[39]	France	Ducks	Free-range + confined	46
[48]	France	Ducks	Free-range	127
[49]	United States of America	Broilers, layers, Turkeys, Ducks, Guinea fowls, Pigeons, game birds**	*Not retrievable*	*NA*
[50]	United States of America	Broilers	Free-range + confined	41
[51]	United Kingdom	Broilers, layers, Turkeys, Ducks, Game birds**	Free-range + confined	96
[52]	Italy	Broilers, Layers	Free-range + confined	18
[53]	Canada	Broilers, layers, Turkeys, Ducks, game birds**	Free-range + confined	98
[54]	Australia	Broilers, layers	Free-range + confined	40
[55]	Australia	Broilers, layers	Free-range + confined	40
[56]	Australia	Broilers, layers	Free-range + confined	40
[57]	Australia	Layers	Free-range	41
[58]	Belgium	Broilers, layers, Turkeys, game birds**	Free-range + confined	*NA*
[59]	Belgium, France, The Netherlands, Italy, Spain, Poland, Hungary, Finland, Sweden, Serbia, Portugal and Germany	Broilers, layers	Free-range + confined	22

^*^It was not possible to identify in all these countries in which ones free-range poultry farms were investigated.

^**^It was not possible in these studies to acertain that all species were concerned by free-range access.

^***^Only free-range farms are reported. NA's were introduced when the study included both confined and free-range farms and when the number of free-range farms was not precised.

**Table 2:** **Studies included in the analysis of biosecurity measures specific to free-range poultry: study design, objectives, and conditions of biosecurity assessment**

Article reference	Study design	Specific pathogen or disease targeted	Number of biosecurity measures evaluated	Biosecurity assessment method	Original questionnaire availability	Biosecurity assessment as part of a scoring tool
[42]	Cross-sectional	No	10	Questionnaire and observations*	Available, external link	Yes
[47]	Cohort or longitudinal	*Campylobacter*	4	No	Not fully available	No
[39]	Cross-sectional	No	4	Questionnaire and observations	Available, upon request	No
[48]	Cross-sectional	No	4	Questionnaire and observations	Available, upon request	Yes
[60]	Cross-sectional	No	1	Questionnaire	Available, upon request	No
[50]	Cross-sectional	*Salmonella*, *Campylobacter*, Antimicrobial resistance	3	Questionnaire	Not fully available	No
[51]	Cross-sectional	Avian Influenza	5	Questionnaire and observations	Not fully available	No
[52]	Cross-sectional	No	9	Questionnaire	Available	No
[53]	Cross-sectional	*Campylobacter*	2	Questionnaire	Described in a previous publication	No
[54]	Cross-sectional	No	19	Questionnaire and observations**	Described in a previous publication	No
[55]	Cross-sectional	Avian influenza	27	Questionnaire and observations**	Not fully available	No
[56]	Cross-sectional	Avian influenza	27	Questionnaire and observations**	Not fully available	No
[57]	Cross-sectional	No	28	Questionnaire	Not fully available	No
[58]	Cross-sectional	Avian influenza	1	Questionnaire	Available, upon request	Yes
[59]	Cohort or longitudinal	No	11	Questionnaire and observations*	Available, external link	Yes

* or ** studies using the same questionnaire or observations.

The number of biosecurity measures refers to the items related to free-range that were extracted.

Most studies were cross-sectional (13/15), and did not target any specific disease (8/15). The remainder focused on one or more pathogens or diseases with zoonotic potential: avian influenza (*n* = 4), *Campylobacter* (*n* = 3), *Salmonella* (*n* = 1), and antimicrobial resistance (*n* = 1). With respect to farm sampling, only two studies included exclusively free-range poultry farms. The number of farms per study ranged from two to 127 (median = 41). Most studies (10/15) included at least two types of poultry (usually broilers and layers), while ducks were exclusively targeted in two French studies.

Regarding the methods used, six protocols relied solely on questionnaires, while eight also included on-farm observations (for one study, this information was unavailable). The number of biosecurity questions or items specifically addressing the use of an outdoor range was highly variable (range: 1–27, median = 5) and is further detailed in the following section. In four studies, biosecurity data was computed into a score.

### Aspects of free-range production assessed for biosecurity

**Figure 3:**
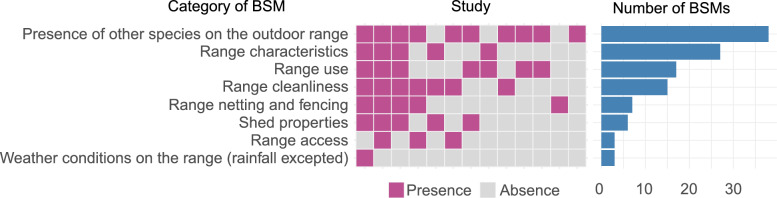
**Number of BSMs reported per study.** The figure presents the number of reported occurrences (*n* = 116) of biosecurity measures (BSMs) specific to free-range poultry farms (blue bars), by BSM category, along with a mapping of the absence (grey colour) or presence (purple colour) of BSM categories across the 15 studies. Only 13 studies are reported here due to studies using the same questionnaire.

**Figure 4:**
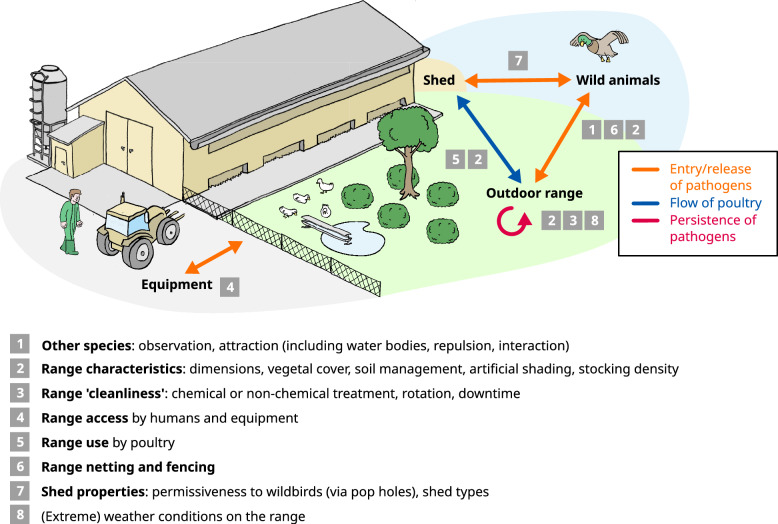
**Representation of pathogen transmission pathways leading to contamination of poultry in free-range systems, along with the categories of biosecurity measures (BSMs) identified in the ScR.**

A total of 116 BSMs were extracted, corresponding to 52 unique BSMs (some were reported in more than one study). It should be noted that some questions or observations covered multiple BSMs (e.g., providing feed and water on the outdoor range was counted as two separate measures). From these 52 BSMs, 23 subcategories and 8 categories were identified. The most frequently assessed or most detailed BSMs concerned the presence of other species (non-poultry) on the outdoor range, particularly wild birds. Other frequently assessed categories related to poultry range use and range cleanliness (e.g., sanitation). A few studies proposed a highly detailed assessment of possible BSMs related to outdoor range use. The number of occurrences of BSMs (from the total of 116) per category is shown in Figure 3, along with the coverage (presence or absence of at least one BSM) of each category across studies. The complete list of BSMs, organized by categories and subcategories with their reported frequency is provided in Tables 3, 4, 5, 6and the complete dataset containing the original item wording used in the articles is provided in Additional file 3. To link these categories to infection routes, we developed a graphical representation of infection pathways and related BSM categories (Figure 4).

**Table 3:** **Categorisation and frequency of biosecurity measures (BSMs) specific to free-range poultry farms, identified in the 15 included studies and associated with the presence of other species on the outdoor range (attraction, interaction and observation)**

Category, subcategory and BSM	Number of occurrences
Presence of other species on the outdoor range: attraction, interaction and observation	
Access to non-poultry domestic animals	
Domestic animals do not have access to the outdoor range	2
Wild bird attraction and interaction mediated by bodies of water	
Average annual rainfall	1
Concrete exit area (to avoid water bodies)	1
Drainage lines running through the outdoor range	1
Topography (slope) of the outdoor range	1
Waterbodies are absent from the outdoor range	5
Waterbodies are not close to the outdoor range	1
Wild bird attraction and interaction mediated by drinkers	
Drinkers are not on the outdoor range	4
Outdoor drinkers are protected from wild birds	3
Wild bird attraction and interaction mediated by feeders or feed on the range	
Eggs are not laid on the outdoor run	1
Feeders are not on the outdoor range	7
Outdoor feeders are protected from wild birds	2
Wild bird observation on the range	
Presence of wild birds on the outdoor range	4
Interaction between poultry and wild birds on the outdoor range	2
Wild non-bird animal observation on the range	
Presence of wild non-bird animals on the outdoor range	2
Interaction between poultry and wild non-bird animals on the outdoor range	1

**Table 4:** **Categorisation and frequency of biosecurity measures (BSMs) specific to free-range poultry farms, identified in the 15 included studies and associated with range characteristics**

Category, subcategory and BSM	Number of occurrences	
Range characteristics		
Range dimensions		
Shape of the range and closeness to the barn	1	
Surface of the outdoor range	2	
Range vegetal cover		
Grass cover (including maintenance)	5	
Grass cover persistence	2	
Irrigation on the range	1	
Shrub cover (including maintenance)	3	
Tree cover (including maintenance)	5	
Tree cover (surrounding the edge of the outdoor range)	1	
Range soil management		
Exit area covered with removable material to avoid mud in the shed	1	
Type of soil	1	
Shade on the range		
Artificial shade structures on the outdoor range	3	
Stocking density on the range		
Stocking density on the range	1	
Other range equipment		
Deliberate range enrichment items (bales and ladders)		1

**Table 5:** **Categorisation and frequency of biosecurity measures (BSMs) specific to free-range poultry farms, identified in the 15 included studies and associated with range cleanliness and access**

Category, subcategory and BSM	Number of occurrences
Range cleanliness	
Rotation and downtime	
Downtime on the outdoor range	1
Same outdoor area not used twice in a row	5
Sanitation	
Application of manure on the outdoor range	1
Chemical treatment of the soil on the outdoor range	4
Non-chemical treatment of the soil on the outdoor range (mowing, manure removal)	4
Range access	
Feed supplier enters the outdoor range	1
No vehicle passage on the outdoor range	1
The visitors use an anteroom (can be the barn anteroom) before accessing the outdoor range	1

**Table 6:** **Categorisation and frequency of biosecurity measures (BSMs) specific to free-range poultry farms, identified in the 15 included studies and associated with range netting and fencing, shed properties, and weather conditions**

Category, subcategory and BSM	Number of occurrences
Range netting and fencing	
Range fencing	
All the outdoor range is correctly fenced off	5
Range netting	
Protective netting on the outdoor range	2
Range use	
Outdoor access calendar	
Minimal age for outdoor range access	4
Outdoor access all the time	3
Range access depending on the weather	1
Range access restrictions depending on epidemiological context	1
Time of the day when outdoor range access is granted	3
Range actual use	
Proportion of poultry on the outdoor range	3
Range actual use	
Proportion of the outdoor range used by poultry	2
Shed properties	
Shed permissiveness	
Proportion of the shed covered by pop-holes	2
Shed run off	
Management of run off from the sheds (collection, treatment)	1
Shed type	
Shed type (fixed or mobile)	3
Weather conditions on the range (rainfall excepted)	
Extreme weather conditions	1
Maximum temperatures	1
Minimum temperatures	1

## Discussion

This ScR identified and categorized biosecurity measures specific to free-range poultry systems and described the contexts in which they were assessed. By mapping and classifying these measures into a structured framework of categories and subcategories, it highlights both the most frequently addressed aspects and the critical gaps, providing a basis for critical reflection on current assessment practices and for guiding the design of future protocols tailored to free-range production systems.

To date, this is the first attempt to explore in detail the possible approaches to assessing biosecurity in free-range poultry systems. Although the conditions of on-farm biosecurity assessment have been recently described in European and African countries, these studies did not specifically address free-range poultry farms [12, 13, 24]. We therefore believe that most biosecurity assessment tools fail to capture the specificities of emerging alternative poultry production systems, specifically with regard to the use of an outdoor range.

In the corpus of publications analyzed, the most frequent category of biosecurity measures concerned the presence of other animal species (mostly birds) on the outdoor range: we counted 13 types of BSM related to the presence (and the interaction with) wild birds on ranges. The fact that wild birds may serve as reservoirs receives considerable attention, probably because of their documented role in the long-distance propagation of avian influenza viruses by migrating birds, particularly waterfowl and shorebirds [25]. Therefore, much focus has been placed in observing wild birds on poultry farms and outdoor ranges, using direct observations or camera traps [26–29], providing insights into the dynamics of wild bird activity on outdoor ranges. However, it is debatable whether observing wild birds on the outdoor range is truly a biosecurity measure per se, since it does not involve any actual infrastructure management or specific action aimed at reducing a risk. For this reason, many biosecurity assessment protocols focus on factors that may attract wild birds on outdoor ranges, such as bodies of water (and feed sources), as illustrated by the 11 different BSMs addressing this topic.

For domestic poultry to be contaminated on outdoor ranges, the actual presence of poultry on the ranges is necessary, and the more intense the exposure, the higher the risk of contamination. This explains why one of the most frequent type of BSM reported was range use. This was assessed using different types of parameters: surface area used by poultry, time spent on the range (daily or cumulative), or proportion of the flock present outdoor. However, some important factors influencing range use by poultry, such as the type and richness of vegetal cover or the presence of artificial shading, were seldom considered (range characteristics were detailed in only 5/15 studies). Behavioural studies have shown that the greater the amount of vegetation or artificial cover [30, 31], the more poultry used the range, as covered areas are cooler in sunny conditions and also provide protection from flying predators. More generally, improving the description of outdoor ranges would help contextualize both their capacity to attract wild birds and their actual range use. Surprisingly, forbidding access to outdoor ranges during (avian influenza) epidemics was reported only once; one might argue that in such cases poultry can no longer be considered free-range. In a recent European survey conducted among free-range pig and poultry farmers, the most important concern (in relation to disease occurrence) was the risk of being forced to stop outdoor farming [32]. Moreover, preventing the use of the outdoor range could also introduce new biosecurity risks: for instance, bedding would require more frequent mulching, and therefore increasing the frequency with which mulching equipment crosses the line of separation between the barn and the rest of the farm site.

The reduction of outdoor range contamination was the third most frequent type of BSM addressed (7/15 studies). We counted multiple occurrences for measures aiming at reducing the infectious pressure on the outdoor range, either by applying treatments (chemical or non-chemical) or by increasing downtime. Concerning downtime, it has been reported that the load of infectious particles decreases over time in an outdoor environment [33].

Surprisingly, some biosecurity measures related to outdoor ranges were very rarely included in the corpus of studies. For example, proper fencing (to prevent interaction with mammals) or netting (to prevent interaction with wild birds) was rarely addressed. Nets are highly effective in preventing birds from accessing to the outdoor range, although very small birds (e.g., passerines), contaminated droppings, or feathers may still pass through depending on mesh size. However, in free-range poultry production settings, considering the “usual” range size (e.g., according to EU organic farming standards in layer hens, range size may reach up to 1.2 hectares), netting the entire range is considered to be too expensive and would require to have specific, smaller tractors. Completely netted outdoor ranges are encountered most exclusively in game bird production, where they are used to prevent birds from escaping. Nonetheless, netting remains an affordable BSM (and should be considered for biosecurity assessments) when regulations allow the use of a “restricted” free-range area under nets during high-risk influenza periods [34, 35]. To reduce the presence of wild birds on outdoor ranges, some repelling practices can also be implemented. Surprisingly, such measures were not referenced in the selected studies. Repelling methods classically involve sounds or scarecrows but shows limited efficacy [36]. However, a recent work—not included in our review—tested the efficacy of laser beams to scare wild birds on the outdoor range of a Dutch poultry farm and demonstrated its capacity (reduction of wild birds visits by 98.2%) to repel several orders of wild birds, including waterfowl. The laser used in this study was a rotating, automated commercial device designed for agricultural purposes and placed on top of a tower within the outdoor range [37]. The lasers are considered as a physical threat by birds and do not cause injury to their eyes.

Our ScR showed that most studies were conducted within the framework of avian influenza or *Campylobacter* research. Parasitic diseases received little attention, which may explain the limited focus on range management practices aimed at controlling parasite intermediate hosts and resistance forms (e.g., oocysts) in the soil. This may originate from the fact that parasite infections are often subclinical rather than acute, with the exception of *Histomonas meleagridis* in turkeys [5, 7, 38], and therefore capturing less attention.

It would be valuable for future studies on biosecurity in free-range poultry flocks to also consider indirectly related BSMs. Free-range poultry farms are usually part of a wider official or unofficial quality scheme (e.g., organic farming, small-scale short food supply chains, integrated companies), which include requirements that can have an effect on on-farm biosecurity. For example, farms in short food supply chains may not follow all-in/all-out flock management and may instead operate multiple small barns (making compartmentalization and anteroom management more difficult to manage) [39, 40].

Compared to BSMs in indoor poultry systems, BSMs in outdoor poultry systems may be perceived by farmers as less effective. Farmers often feel powerless against the risk of infection posed by wild birds, believing they have limited ability to prevent wild bird interactions with domestic poultry, even in indoor systems [41]. Although two studies attempted to weigh the relative importance of BSMs specific to free-range systems, we still lack evidence on their relative efficacy and potential return on investment of each of BSM identified in this ScR [42].

This study shows potential limitations. Some BSMs related to free-range poultry systems may have been missed for several reasons. Some research articles may have been overlooked due to the inclusion criteria. We used a criterion based on “high-income” countries, which is difficult to define rigorously and may have excluded some valuable production systems and related articles. In addition, we focused on protocols published in scientific studies, while some biosecurity assessment protocols are used in public veterinary health and industry settings and these are not publicly available [12, 13, 43]. However, due to probable limited access to industry documents or language barriers, the scientific literature still provided a rich source of information. Much of the available literature on free-range poultry farms emphasises poultry welfare and behaviour rather than biosecurity and disease [30, 44, 45]. It would therefore be worth for future research to identify and analyze potential conflicts between biosecurity measures and welfare issues in free-range production systems, as previously attempted for some indoor productions [46].

In conclusion, we provide here a catalogue, classification and discussion of BSMs specific to free-range poultry systems. However, rather than proposing a standardized checklist for biosecurity assessment, we invite readers to acknowledge the wide spectrum of measures that we documented and to adapt their protocols according to their specific needs.

## Supplementary Information

**Additional file 1. Bibliographic search strategy used to identify studies assessing biosecurity measures in free-range poultry farms in high-income countries.**

**Additional file 2. Article description and characteristics.** Table presenting full article description and main characteristics, as extracted by the authors.

**Additional file 3. List and classification of biosecurity measures related to the use of an outdoor range.** Table presenting the detail of the questions on biosecurity measures associated with the use of an outdoor range (observations from checklists are considered in this table as questions), as extracted from the articles, along with the associated BSM, the subcategory and the category. The reference of the article is given (numbers). The correspondence between article number and article description is given is Additional file 2.

## Data Availability

The datasets supporting the conclusions of this article are included within the article and its additional files.
