# Supplementary material for: Which biosecurity measures are specific to free-range poultry? Insights from a scoping review
Source: Vet Res. 2026 Apr 2;57:65. doi: 10.1186/s13567-026-01742-w (PMC13154871; doi:10.1186/s13567-026-01742-w)
Supplement: Supplementary file 1 — Additional file 1. Bibliographic search strategy used to identify studies assessing biosecurity measures in free-range poultry farms in high-income countries. [file 13567_2026_1742_MOESM1_ESM.docx]

| **Major terms** | **keywords** |
| --- | --- |
| #1 Biosecurity | biosecurity OR bio-security |
| #2 Free-range | extensive OR free-range OR “free range” OR outdoor OR organic OR small-scale OR “small scale” OR “small holder” OR open-air OR “open air” OR pasture* OR yard* |
| #3 Farm | farm* |
| #4 Poultry | poultry OR fowl* OR avian* OR bird* OR chick* OR broiler* OR layer* OR hen* OR gallus OR breeder* OR duck* OR anas OR turkey* OR meleagri* OR flock* OR livestock OR “food producing animal*” OR “food-producing animal*” OR “food animal*” OR “animal husbandry*” OR “domestic animal*” |
| #5 Assessment | assess* OR risk* OR level* OR evaluation* OR adopt* OR implement* OR measure* OR scor* OR questionnaire* OR checklist* OR benefit* OR consequence* OR impact OR practice* OR compliance |
| #6 High-Income countries | Europe* OR “European Union” OR EU OR Austria* OR Belgium OR Bulgaria* OR Croatia* OR Cypr* OR Czech* OR Denmark OR Estonia OR Finland OR France OR German* OR Gree* OR Hungar* OR Iceland OR Ireland OR “Irish Republic” OR Ital* OR Kosovo OR Latvia OR Lithuania* OR Luxembourg OR Malt* OR Montenegro OR Macedonia* OR Netherlands OR Norway OR Poland OR Portug* OR Romania* OR Serbia* OR Slovakia OR Slovenia* OR Spain OR Sweden OR Belarus OR Moldova OR Bosnia and Herzegovina OR Ukraine OR Andorra OR Liechtenstein OR Monaco OR Switzerland OR “United Kingdom” OR UK OR England OR Scotland OR Wales OR “United States” OR “United States of America” OR US OR USA OR Canada OR Australia OR “New Zealand” OR Japan OR Russia* OR Soviet OR USSR |
| #2 AND #3 | records screened |
| #1 AND #4 AND #5 AND #6 AND #7 | records screened |
